# Effect of single intravenous injection of esketamine on postpartum depression after labor analgesia and potential mechanisms: a randomized, double-blinded controlled trial

**DOI:** 10.1186/s40360-023-00705-7

**Published:** 2023-11-23

**Authors:** Bin Ling, Yun Zhu, Zelin Yan, Hao Chen, Hua Xu, Qi Wang, Wanyou Yu, Wei Wang

**Affiliations:** 1 Department of Anesthesiology, the Affiliated Jiangning Hospital of Nanjing Medical University, CN-Jiangsu, No. 169 Hushan Road, Nanjing, 211100 China; 2 Department of Anesthesiology, Nanjing Jiangning Hospital of Traditional Chinese Medicine, Nangjing, 211100 China; 3 Department of Gynaecology and obstetrics, the Affiliated Jiangning Hospital of Nanjing Medical University, Nangjing, 211100 China

**Keywords:** Esketamine, Depression, Postpartum, Analgesia, Obstetrical

## Abstract

**Background:**

The study was designed to investigate effects of single intravenous injection of esketamine on the incidence of postpartum depression (PPD) after labor analgesia and explore the potential mechanisms.

**Methods:**

A total of 120 women who underwent labor analgesia by epidural analgesia pump were enrolled and divided into two groups randomly. Esketamine at a dose of 0.2 mg/kg was intravenously injected after fetal disengagement in the test group and placebo was administered in the control group. The occurrence of PPD and side effects after delivery were recorded. Some indicators related to stress and inflammation were measured before labor analgesia and at 24 h, 1 week, and 6 weeks after delivery in this study. Data were analyzed by independent t-test, repeated measures analysis of variance and Chi-square test in SPSS software (version 25.0). It was considered statistically significant since a p value less than 0.05.

**Results:**

The incidence of PPD was significantly decreased both for one week and six weeks after delivery by using of esketamine (3.4% vs. 15.3%, p = 0.004 and 5.2% vs. 18.6%, p = 0.006, respectively). There were also significant differences between the stress and inflammation-related indicators in different time points in this study, while the side effects for 48 h after delivery were similar between the two groups.

**Conclusions:**

Single intravenous injection of esketamine after delivery in participants underwent labor analgesia can decrease the occurrence of postpartum depression for one week and six weeks after delivery, while the side effects were not increased. The antidepressant effects of esketamine may be related to the reduction of stress response and inflammation.

**Trial registration:**

The trial was registered at the Chinese Clinical Trial Registry on 5/30/2022 (CTRI registration number—ChiCTR2200060387). URL of registry: https://www.chictr.org.cn/bin/home.

**Supplementary Information:**

The online version contains supplementary material available at 10.1186/s40360-023-00705-7.

## Introduction

Due to the particularity of physiology, maternal perinatal safety has been a special concern. At present, the labor analgesia is gradually recommended on the premise of ensuring maternal and fetal safety [1]. Postpartum depression (PPD) is a mental illness occurring in the puerperium, showing a series symptoms such as depression, sadness, crying, irritability, even hallucinations or suicide, which seriously endangers the physical and mental health of pregnant women [2, 3]. PPD is related to maternal psychological and physical, family, social and obstetric factors, and occurs during six weeks after delivery in either primigravida or multipara undergoing cesarean section or vaginal delivery [4, 5], with the incidence even up to 38.5% [6]. Due to its high incidence and harmfulness, the treatment and prevention of PPD have become an important topic.

Nowadays, medication combined with psychological counseling are mainly used for the treatment of PPD. However, long-term medication may have side effects for lactating women on the infant’s motional, behavioral and neurological development [7]. Thus, prevention of PPD is even more important than treatment. As an intravenous anesthetic drug, ketamine is often used in pediatric, obstetric, and outpatient anesthesia. In addition, as a psychotropic drug, the antidepressant effect of ketamine is also gradually recognized. Studies have shown that ketamine can significantly reduce the incidence and severity of depression [8, 9]. As an isomer of ketamine, compared with racemic ketamine, esketamine has stronger analgesic and sedative effects, smaller cardiovascular and psychiatric adverse effects, and can also be used in patients with refractory, severe depression and epilepsy [10]. Our previous studies have suggested that esketamine prevent PPD in women underwent cesarean Sects. [11, 12]. However, with the widespread use of labor analgesia in obstetrics, it was also unknown whether esketamine was effective in such maternal postpartum depression. Additionally, the mechanisms of antidepressant effects of esketamine were unclear now. Therefore, we designed the study which was aimed to confirm the antidepressant effect of esketamine in women underwent labor analgesia and explore potential mechanisms.

## Methods

### Study design

This randomized, double-blinded, controlled trial was conducted at the Affiliated Jiangning Hospital of Nanjing Medical University, Nanjing, China, from 6/1/2022 to 2/28/2023, which performed in accordance with the declaration of Helsinki. The Consolidated Standards of Reporting Trials (CONSORT) recommendations were followed in this study [13]. The study protocol was approved by the medical ethics committee of our hospital on 1/14/2022 (Ethical approval number: 2021-03-031-K01). Then, the study was registered at the Chinese Clinical Trial Registry on 5/30/2022 (CTRI registration number—ChiCTR2200060387). All participants involved were informed of the proposal and gave their written, informed consent form voluntarily before enrollment.

### Study participants

One hundred and twenty primiparous women who underwent labor analgesia, aged 22 to 38, with American society of Aneshesiologists (ASA) physical status II, with 1 fetal 0 labor, pregnancy time greater than 38 weeks and less than 42 weeks, and single term pregnancy, were enrolled. The exclusion criteria were as follows: not suitable for transvaginal delivery; combined with coagulation disorders; having mental disorder; organic or pharmacogenic depression before delivery; the Self-rating Anxiety Scale (SAS) ≥ 50 or the Self-rating Depression Scale (SDS) ≥ 0.5 [14]; perineal tears or a lateral cut required during delivery; Apgar score of fetus at delivery < 8; postpartum hemorrhage > 500 ml; failure of epidural puncture; changed to cesarean section.

### Sample size estimation

**Fig. 1:**
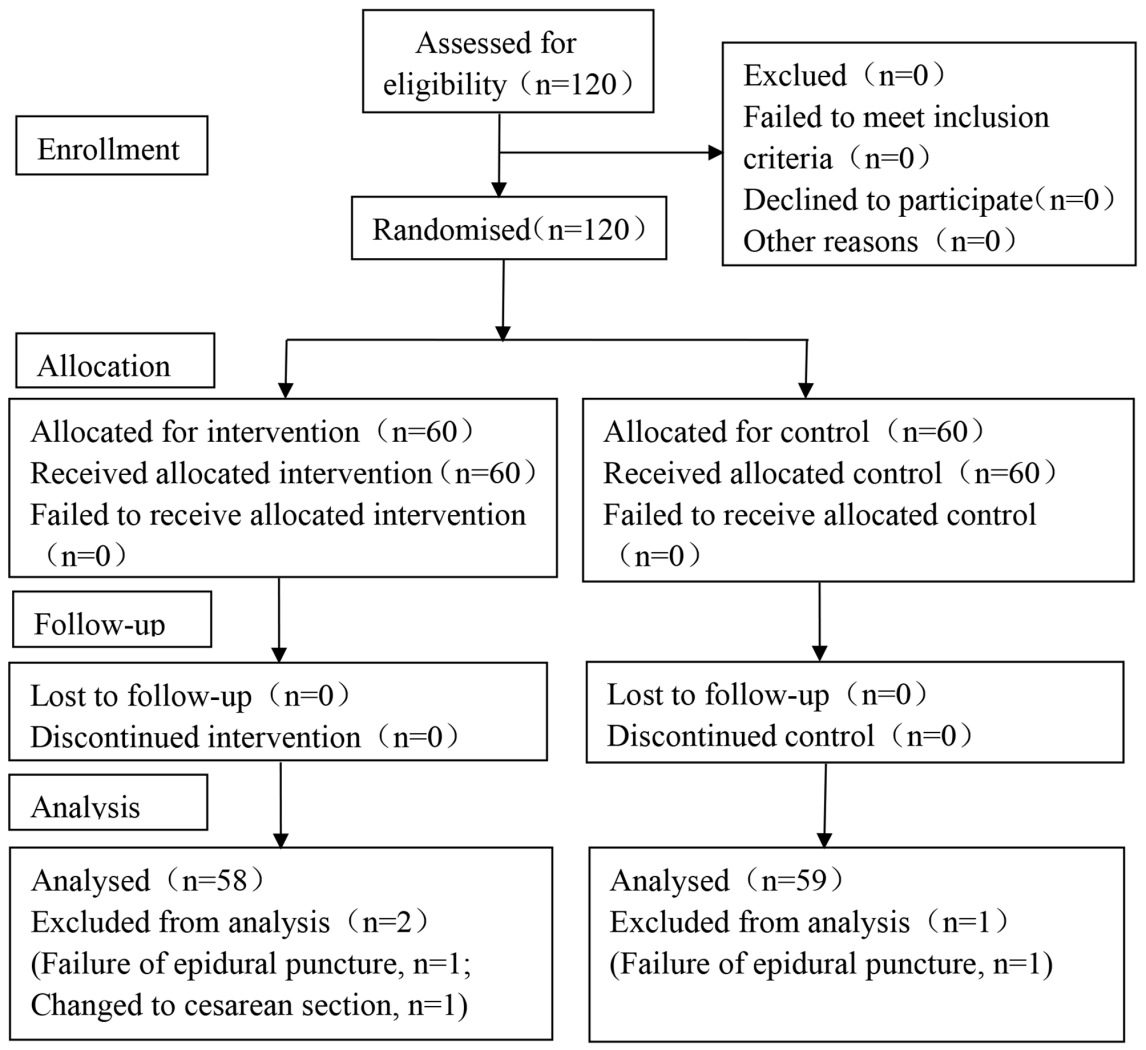
CONSORT flow diagram. In the study, a total of 120 participants were enrolled initially and three participants were excluded during the trial. Finally, There were 117 participants included in the statistical analysis (58 in Group E and 59 in Group C, respectively). CONSORT: the Consolidated Standards of Reporting Trials

Based on the results of our pre-experiment (10 participants in each group), the incidence of PPD at 6 weeks after delivery can be reduced by 12% in the tested group. Power analysis showed that a reduction rate of 15% with *α* = 0.05 and a 10% dropout rate within a power value of 90%, a sample size of at least 54 per group were needed. A total of 120 samples were designed in this study, for 60 in each group respectively. Figure 1 showed the consolidated standards of reporting trials (CONSORT) flow diagram of the study participant’s recruitment.

### Randomization and allocation concealment

Participants were randomly assigned to one of two groups. Random tables were generated by SPSS 20.0. One hundred and twenty sealed envelopes were prepared by a statistician who did not participate in the study. The study was performed with neither patients’ nor observers’ awareness of the group to which each patient belonged. To assure concealment of allocation, numbers were kept in sealed and opaque envelopes, which were opened by an anaesthesiologist who was not involved in the study.

### Study interventions

After entering the delivery room, peripheral venous access was opened in all participants, maternal heart rate, noninvasive blood pressure measurement, electrocardiography and pulse oximetry were monitored. All women were received oxygen for 3 L/min by transnasal catheter. Epidural anaesthesia were operated between L_2_ and L_3_ after cervical dilation up to 2 ~ 3 cm. After successful puncture, the epidural catheter was placed for 3.5 cm toward the head position and 1% lidocaine was injected for 3 ml. The epidural analgesia pump was connected, in which was 0.75% ropivacaine hydrochloride (20 ml) were diluted by normal saline up to 100 ml. All pumps were set with a bolus of 10 ml, continuous infusion amount of 8 ml/h, single dose of 4 ml, locking time of 15 min and stopped after fetal disengagement. Esketamine (0.2 mg/kg) was intravenously injected after fetal disengagement in the test group (Group E), while the placebo (normal saline) was administered in the control group (Group C). Labor analgesia was performed by the same anesthesiologist and esketamine or placebo were administrated by the same nurse anesthetist. Perinatal management was completed by the same obstetric team. Neither the subjects and the investigators were aware of the enrollment.

### Outcome measurements

The primary outcomes of the study were the incidence of PPD at one week and six weeks after delivery. The diagnosis of postpartum depression was based on the reference [15] and [16], in which the depression score (Edinburgh Postpartum Depression Scale, EPDS) and the diagnostic criteria for mental disorders are included.

Ramsay sedation score before analgesia (T_0_), 5 (T_1_), 10 (T_2_), 20 (T_3_), 30 (T_4_) and 60 (T_5_) minutes after administration of esketamine were measured. The scoring criteria [17] were as follows: 1 point, patients were awake, feeling anxiety or irritability; 2 points, patients were awake, being cooperative, directional or quiet; 3 points, patients were awake, only being responsive to commands; 4 points, patients were asleep, being responsive quickly to tapping eyebrow or soft tone stimulation; 5 points, patients were asleep, being responsive slowly to tapping eyebrow or strong acoustic stimulation; 6 points, patients were asleep, being no responsive to tapping eyebrow or strong acoustic stimulation.

All women were tested levels of norepinephrine (NE), epinephrine (E) ,C-reactive protein (CRP), interleukin-6 (IL-6) and interleukin-10 (IL-10) in peripheral venous blood before labor analgesia (when the woman was quiet in the ward), at 24 h, 1 week, and 6 weeks after delivery. Serum concentrations of IL-6 and IL-10 were determined by enzyme linked immunosorbent assay (ELISA).

The duration of the first and second stage of labor, the Apgar score of fetus at delivery, and the volume of postpartum hemorrhage was calculated for 24 h after delivery. Side effects such as nightmares, drowsiness, nausea and vomiting were measured for 48 h after delivery as well.

### Statistical analysis

The statistical analysis were performed using SPSS software package (version 25.0; IBM, Inc., Chicago, IL, USA). The continuous data were presented as mean (M) ± standard deviation (SD) and examined by independent t-test. The categorical data were shown as n(%) and examined with a Chi-square test. It was considered statistically significant since a p value < 0.05.

## Results

### Patient recruitment

In the study, a total of 120 participants were enrolled initially and three participants were excluded during the trial. One case was excluded for failure of epidural puncture, and another one was for changed to cesarean section in Group E, while one participant was excluded for failure of epidural puncture in Group C. Finally, There were 117 participants included in the statistical analysis (58 in Group E and 59 in Group C, respectively. Figure 1, CONSORT flow diagram).

### Patient characteristics

There were no significant difference of the patient characteristics (age, height, weight, BMI, gestational week, the duration of the first and second stage of labor, the Apgar score of fetus at delivery, volume of postpartum hemorrhage) between the two groups (Table 1).

**Table 1:** Baseline characteristics of the studied groups

Characteristic	Group E (n = 58)	Group C (n = 59)	*P-*value ^a^
Age (years)	28.2 ± 4.8	27.8 ± 4.4	0.656
Gestational age (weeks)	38.8 ± 3.7	39.0 ± 2.6	0.338
Height (cm)	159.2 ± 6.7	157.8 ± 6.2	0.419
Weight (kg)	67.8 ± 7.1	66.9 ± 5.9	0.813
BMI (kg/m^2)^	28.2 ± 4.1	27.8 ± 3.6	0.249
Duration of the first stage of labor (min)	576.3± 91.2	591.6± 96.4	0.676
Duration of the second stage of labor (min)	104.6 ± 13.3	101.9 ± 11.8	0.466
Apgar score of fetus at delivery (points)	9.0 ± 1.2	9.1 ± 1.3	0.798
Postpartum hemorrhage (ml)	216.5 ± 33.8	202.3 ± 39.2	0.825

Data presented as mean ± Standard Deviation.

BMI: Body mass index.

^a^ Independent sample t-test.

### Ramsay sedation score

There were no significant difference of the Ramsay sedation score at T_0_ and T_5_ between the two groups. Compared with Group C, the Ramsay sedation score at T_1_, T_2_, T_3_ and T_4_ were significantly higher in Group E (Table 2).

**Table 2:** Ramsay sedation score in the two groups

Group	T_0_	T_1_	T_2_	T_3_	T_4_	T_5_
Group E (n = 58)	1.65 ± 0.38	2.48 ± 0.45	2.57 ± 0.51	2.64 ± 0.49	2.58 ± 0.52	1.72 ± 0.43
Group C (n = 59)	1.71 ± 0.44	1.85 ± 0.41	2.07 ± 0.43	2.12 ± 0.38	2.08 ± 0.42	1.75 ± 0.40
*P-*value ^a^	0.218	0.001	0.003	0.006	0.005	0.556

Data presented as mean ± Standard Deviation

^a^ Independent sample t-test

### Incidence of PPD and side effects

The incidence of PPD was significantly decreased both for one week and six weeks after delivery in Group E (3.4% vs. 15.3%, p = 0.004 and 5.2% vs. 18.6%, p = 0.006, respectively).The incidence of side effects after delivery were similar between the two groups (Table 3).

**Table 3:** Occurrence of PPD and side effects in the two groups

Group	PPD		nausea, vomiting	drowsiness	nightmares
	1 week	6 weeks			
Group E (n = 58)	2(3.4)	3(5.2)	2(3.4)	1(1.7)	2(3.4)
Group C (n = 59)	9(15.3)	11(18.6)	1(1.7)	1(1.7)	3(5.1)
*P-*value ^a^	0.004	0.006	0.695	0.784	0.815

Data presented as number (percentage)

PPD: postpartum depression

^a^ Chi square

### Levels of NE, E, CRP, IL-6 and IL-10

There were no significant difference of the levels of NE, E, CRP, IL-6 and IL-10.

between the two groups before labor analgesia and 6 weeks after delivery. Compared with Group C, the levels of NE, E, CRP and IL-6 were significantly lower for 24 h and 1 week after delivery in Group E, while IL-10 were higher at the same time (Table 4).

**Table 4:** Levels of NE, E CRP, IL-6 and IL-10 in the two groups

Variables	Group	Before analgesia	24 h after delivering	1st week after delivering	6th week after delivering
NE (nmol/L)	Group E (n = 58)	4.36 ± 0.77	3.11 ± 0.54	2.95 ± 0.49	3.06 ± 0.54
	Group C (n = 59)	4.58 ± 0.84	4.24 ± 0.71	4.15 ± 0.73	3.31 ± 0.61
*p* value		0.629*	0.009*	0.004*	0.428*
E (pmol/L)	Group E (n = 58)	526.3 ± 38.6	367.2 ± 29.1	339.4 ± 24.7	326.5 ± 23.3
	Group C (n = 59)	549.8 ± 44.5	481.7 ± 37.4	477.9 ± 33.6	341.1 ± 28.6
*P-*value ^a^		0.858	0.001	0.006	0.359
CRP (mg/L)	Group E (n = 58)	2.52 ± 0.69	7.87 ± 1.38	6.35 ± 1.12	2.84 ± 0.81
	Group C (n = 59)	2.47 ± 0.75	10.98 ± 1.76	8.27 ± 1.33	3.02 ± 0.94
*P-*value ^a^		0.228	0.008	0.005	0.656
IL−6 (ng/L)	Group E (n = 58)	78.8 ± 9.6	145.6 ± 12.1	108.4 ± 11.2	81.5 ± 8.7
	Group C (n = 59)	76.9 ± 8.7	186.2 ± 15.6	141.3 ± 12.6	79.8 ± 9.1
*P-*value ^a^		0.326	0.007	0.002	0.526
IL−10 (µg/L)	Group E (n = 58)	15.2 ± 5.3	8.7 ± 3.4	10.7 ± 5.2	13.9 ± 6.1
	Group C (n = 59)	16.7 ± 6.5	5.1 ± 2.2	6.9 ± 3.1	15.1 ± 6.7
*P-*value ^a^		0.916	0.004	0.005	0.778

Data presented as mean ± Standard Deviation

NE: norepinephrine; E: epinephrine; CRP: C-reactive protein; IL-6: interleukin-6; IL-10: interleukin-10

^a^ Independent sample t-test

## Discussion

Pain in the perinatal period is one of the factors leading to the development of PPD. The symptoms of PPD are more prominent in women who experience frequent pain during childbirth [18]. Although maternal pain can be relieved by labor analgesia, psychological degeneration, emotional vulnerability, stress response and inflammation may also lead to a high occurrence of PPD [19]. The study of Bruno et al. [20] also showed that anger experience and expression can be considered as vulnerability factors for postpartum mood disorders onset. In the study of Rizzo et al. [21], the role of subthreshold mental disorders were examined as predictors of PPD. The results suggested that agoraphobia/panic, depressed mood, social anxiety and eating problems relate positively to PPD at 3/6 months. In our study, participants combined with coagulation disorders, mental disorder; organic or pharmacogenic depression, SAS ≥ 50 and SAS ≥ 0.5 before delivery were all excluded. The results of this study showed that single intravenous injection of esketamine decreased the occurrence of PPD for one week and six weeks after delivery in women underwent labor analgesia without increasing related side effects.

The traditional treatment for depression is to increase the amount of serotonin in the synaptic space to achieve the effect of alleviating depression, but these antidepressants are slow to act, generally taking three to six weeks to achieve therapeutic effects [22]. In 2019, the U. S. Food and Drug Administration (FDA) granted esketamine for the treatment of major depressive disorder and drug-resistant depression with urgent suicide risk. The results of a meta-analysis indicated that esketamine can significantly improve the patients’ low mood, low self-evaluation, and even weaken the suicidal ideation, especially improve the therapeutic effect for treatment resistant depression (TRD) [23].

Another retrospective analysis have showed that both esketamine’s approval for use in TRD and longer-term safety data may position it preferentially above racemic ketamine, though the efficacy of esketamine compared to racemic ketamine remained unclear [24]. However, there are few studies on esketamine used in postpartum depression. Our previous studies indicated that the incidence of PPD was reduced by prophylactic intravenous use of esketamine or combined with patient control intravenous analgesia for women underwent cesarean Sects. [11, 12]. The study of Wang et al. [25] also have showed that the application of esketamine after cesarean section can effectively reduce depression-related scores and the risk of postpartum depression without increasing adverse reactions and had high safety. However, a lower dose of esketamine was used in our study. Based on previous studies, we applied esketamine to the prevention of postpartum depression in labor analgesia and achieved certain results in this study.

As endocrine hormones, NE and E can affect the bodies’ mood by modulating the sensitivity of neuronal synapses and neurotransmitters [26]. NE can return to the synaptic space under the action of NE transporter protein, and also form E by acting on phenylmethylamine-N-methyltransferase, which easily lead to fear and tension [27]. It had been reported that the blue spot located in the brain stem was activated due to the changes in stress and hormone levels during pregnancy, thus releasing a large number of NE to participate in the regulation of the nervous system, which was easy to cause tension and depression [28]. In this study, the levels of NE and E were significantly lower at 24 h and 1 week after delivery in Group E than in Group C, which also suggested that the maternal stress response was decreased in the tested group. The antidepressant mechanism may be the target of esketamine for monoaminergic nervous system, can affect the brain stem blue spot, and further affect the noradrenergic system, promote the transcription and expression of the gene, increase the synthesis of transporter, resulting in NE migration from plasma membrane to cytoplasm, decrease the concentration of serum NE and E [29]. Additionally, due to the sedative and analgesic effects of esketamine, the Ramsay sedation score was decreased at 5, 10, 20 and 30 min after administration of esketamine in this study, which might be one of the reasons for the reduced stress response in the tested group.

As a non-specific inflammatory marker, CRP reflects the stress and inflammation response of the human body. When infection and injury take place, CRP rises sharply in the plasma [30]. Besides, among the inflammatory mediators released by the human body under pain and traumatic stress, IL-6 is the earliest and highly sensitive proinflammatory factor expressed, which is closely related to the size of trauma, body immune status and prognosis [31]. IL-10 is an inflammatory suppressor, which can exert an anti-inflammatory effect by inhibiting nuclear factors activity and promoting neutrophil apoptosis to reduce pro-inflammatory factors [32]. Therefore, the levels of CRP, IL-6 and IL-10 were observed in the present study to measure the effects of esketamine on inflammatory response. In this study, the levels of CRP and IL-6 were significantly lower at 24 h and 1 week after delivery in the tested group, while IL-10 were higher at the same time. This results indicated that the antidepressant effect of esketamine might be related to the reduced inflammatory response in puerperants underwent labor analgesia.

Studies have shown the brain causes negative emotions by the clustering of the lateral halterate nucleus, where the excitatory transmitter is the N-methyl-D-aspartate (NMDA) receptor of the glutamate-gated ion channel [33, 34]. As a blocker of the glutamate-gated ion channel NMDA receptor, the main antidepressant mechanism of esketamine is to sustainable block the NMDA receptor, block the elongation factor 2 (eEF 2) kinase, increase the release of brain-derived neurotrophic factor (BDNF) and the expression of tropomyosin receptor kinase B (TrkB), induce rapamycin target complex 1 (mTORC1) signaling pathway and extracellular regulatory protein kinase (ERK) activation, improve neuroplasticity and synapse formation [35, 36]. In short, the traditional treatment of antidepression drugs is to stimulate the “reward center” of the brain, while esketamine is to remove the inhibitory effect of the “reward center”.

Esketamine has been used clinically for many years as an anesthetic drug, which is now also widely concerned as a new antidepressant. Considering the possible side effects such as nightmare, hallucination and abusement [37], esketamine was used only once in the study and was lower than the subanesthetic dose. In addition, through the follow-up after delivery, we found that the adverse reactions did not increase in the test group.

There were some limitations in this study, such as the strong subjectivity of the PPD evaluation by EPDS. To minimize the subjective differences, the PPD valuator in this study was the same physician who did not participate in the specific study. Furthermore, the objective indicators such as levels of, NE, E, CRP, IL−6 and IL−10 were also combined in the study, which could better reflect maternal depression. Furthermore, the severity of PPD was not measured in this study, which would be content of next studies.

## Conclusions

In conclusion, single intravenous injection of esketamine (0.2 mg/kg) after delivery in participants underwent labor analgesia can decrease the occurrence of postpartum depression for one week and six weeks after delivery, while the side effects were not increased. The antidepressant effects of esketamine may be related to the reduction of stress response and inflammation.

### Electronic supplementary material

Below is the link to the electronic supplementary material.

Supplementary Material 1

## Data Availability

The datasets used and analyzed during the current study are available from the corresponding author on reasonable request. Wei Wang, e-mail: wangwei2024@163.com.

## Abbreviations

PPD: postpartum depression
ASA: American society of Anesthesiologists
CONSORT: consolidated standards of reporting trials
SAS: the Self-rating Anxiety Scale
SDS: the Self-rating Depression Scale
EPDS: Edinburgh Postpartum Depression Scale
NE: norepinephrine
E: epinephrine
CRP: C-reactive protein
IL-6: interleukin-6
IL-10: interleukin-10
NMDA: N-methyl-D-aspartate
