# Supplementary material for: Effect of single intravenous injection of esketamine on postpartum depression after labor analgesia and potential mechanisms: a randomized, double-blinded controlled trial
Source: BMC Pharmacol Toxicol. 2023 Nov 23;24:66. doi: 10.1186/s40360-023-00705-7 (PMC10668401; doi:10.1186/s40360-023-00705-7)
Supplement: Supplementary file 1 — Supplementary Material 1 [file 40360_2023_705_MOESM1_ESM.doc]

**Research protocol**

**Project summary**:

**Objective:** This is a randomized, double-blinded controlled trial followed the Consolidated Standards of Reporting Trials (CONSORT) recommendations. The aim of the study is to evaluate the effect of single intravenous injection of esketamine on the occurrence of postpartum depression (PPD) after labor analgesia and explore the potential mechanisms.

**Methods:** A total of 120 primiparous women who underwent labor analgesia from June 1st 2022 to February 28th 2023 in the Affiliated Jiangning Hospital of Nanjing Medical University were enrolled, with single term pregnancy, ASA II, aged 22 to 38. According to whether esketamine was administered, all participants were randomly divided into esketamine group (Group E) and control group (Group C) . Epidural anaesthesia were operated in all women between L_2_ and L_3_ after cervical dilation up to 2 ~ 3 cm. The epidural analgesia pump was connected in which was 0.75% ropivacaine hydrochloride (20 ml) diluted by normal saline up to 100 ml. Esketamine (0.2 mg/kg) was intravenously injected after fetal disengagement in Group E and the pump was stopped, while the equal volume of normal saline was administered in Group C. The Ramsay sedation score before analgesia (T_0_), 5 (T_1_), 10 (T_2_) , 20 (T_3_) ,30 (T_4_) and 60 (T_5_) minutes after analgesia were measured. The incidence of PPD was recorded at 1 week and 6 weeks after delivering. The volume of postpartum hemorrhage was calculated for 24 hours and side effects such as nausea and vomiting, drowsiness, and nightmares were also recorded for 48 hours after delivering. The levels of norepinephrine (NE) , epinephrine (E) , C-reactive protein (CRP) , interleukin-6 (IL-6) and interleukin-10 (IL-10) in the peripheral venous blood were measured before labor analgesia and at 24 hours, 1 week, and 6 weeks after delivering.

**Expected results:** Compared with Group C, the Ramsay sedation score at T_1_, T_2_, T_3_ and T_4_ were significantly higher in Group E (*p*<0.01) . Compared with Group C, the incidence of PPD was significantly lower for 1 week and 6 weeks after delivering in Group E (*p*<0.01) . Compared with Group C, the levels of NE, E, CRP and IL-6 were significantly lower at 24 hours and 1 week after delivering in Group E (*p*<0.01) , whlie IL-10 was higher at the same time (*p*<0.01) . There were no significant difference of the side effects for 48 hours after delivering between the two groups(*p*>0.05).

### **General information:**

**Title:** Effect of single intravenous injection of esketamine on postpartum depression after labor analgesia and potential mechanisms: a randomized, double-blinded controlled trial

**Investigators:** Bin Ling, Yanyun Xie, Zelin Yan, Hao Chen, Hua Xu, Qi Wang, Wanyou Yu, Wei Wang. Department of Anesthesiology, the Affiliated Jiangning Hospital of Nanjing Medical University, Nangjing, 211100, China.

Hua Xu, Department of Gynaecology and obstetrics, the Affiliated Jiangning Hospital of Nanjing Medical University, Nangjing, 211100, China

Correspondence: Wei Wang, Department of Anesthesiology, the Affiliated Jiangning Hospital of Nanjing Medical University, No. 169 Hushan Road, CN-Jiangsu, Nanjing, 211100, China, wangwei2024@163.com

**Authors’ contributions:**

**Conceptualization:** Bin Ling, Yayun Xie, Hua Xu, Wei Wang.

**Data curation:** Bin Ling, Qi Wang, Wei Wang.

**Formal analysis:** Bin Ling, Hao Chen, Wanyou Yu.

**Investigation:** Bin Ling, Yayun Xie, Zelin Yan, Hao Chen, Hua Xu, Qi Wang.

**Methodology:** Bin Ling, Yayun Xie, Wanyou Yu, Wei Wang.

**Project administration:** Bin Ling, Wei Wang.

**Supervision:** Wei Wang.

**Validation:** Yayun Xie, Wei Wang.

**Writing – original draft:** Bin Ling, Yayun Xie.

**Writing – review & editing:** Bin Ling, Wanyou Yu, Qi Wang, Wei Wang.

**Rationale & background information:**

Maternal health has always been the focus. With the physical and psychological changes, the incidence of maternal postpartum depression (PPD) is about 3.5%~33% [1]. The causes of PPD are complex and associated to maternal genetic, physiological, psychological, family, social and other factors. Studies have shown that maternal combined diseases, stressing state, disturbed mood, poor sleep quality, pain during pregnancy, disharmonious family relations and lack of social support are the major risk factors for PPD [2, 3]. PPD mainly occurs during 6 weeks after delivering in both primiparous and multiparous women in both vaginal birth and cesarean delivery [4]. The maternal mental state not only affects their own health, but also is very important to the infants and the whole family. Events of suicide and infanticide often have been reported due to PPD [5].

Nowadays, PPD is mainly treated by medication combined with psychological counseling. However, long-term medication may have side effects for lactating women on the infant's neurological, motional and behavioral development [5, 6]. Therefore, prevention of PPD is even more important than treatment. As an intravenous anesthetic drug, ketamine is often used in pediatric, obstetric, and outpatient anesthesia. In recent years, the antidepressant effects of ketamine have been paid more attention in the treatment of mental illness. Studies have showed that ketamine can rapidly improve depressive symptoms and significantly reduce the risk of suicide in patients with resistant depression [7, 8]. Esketamine is an S-Enantiisomer of ketamine and has about twice the affinity to N-methyl-D-aspartate receptor (NMDAR), which is a hot topic of antidepressant research [9]. Our previous studies have suggested that esketamine prevent PPD in women underwent cesarean section [10, 11]. However, with the widespread use of labor analgesia in obstetrics, it was also unknown whether esketamine was effective in such maternal postpartum depression. Additionally, the mechanisms of antidepressant effects of esketamine were unclear now. Therefore, this study was designed to investigate the effect of prophylactic use of esketamine on PPD after labor analgesia and explore potential mechanisms to provide a reference for clinical prevention of PPD.

In this study, some objective indicators such as NE, E, CRP, IL-6 and IL-10 are introduced to analyze their relationship with postpartum depression. As endocrine hormones, NE and E can affect the the bodys’ mood by modulating the sensitivity of neuronal synapses and neurotransmitters [12]. NE can return to the synaptic space under the action of norepinephrine transporter protein, and also form E by acting on phenylmethylamine-N-methyltransferase, which easily lead to tension and fear [13]. It has been reported that due to the changes in stress and hormone levels during pregnancy, the blue spot located in the brain stem was activated, thus releasing a large number of NE to participate in the regulation of the nervous system, which will be easy to cause tension and depression [14]. The antidepressant mechanism may be the target of esketamine for monoaminergic nervous system, can affect the brain stem blue spot, and further affect the noradrenergic system, promote the transcription and expression of the gene, increase the synthesis of transporter, resulting in NE migration from plasma membrane to cytoplasm, decrease the concentration of serum NE and E [15].

As a non-specific inflammatory marker, CRP reflects the stress and inflammation response of the human body. When infection and injury take palce, CRP rises sharply in the plasma [16]. Besides, among the inflammatory mediators released by the human body under pain and traumatic stress, IL-6 is the earliest and highly sensitive proinflammatory factor expressed, which is closely related to the size of trauma, body immune status and prognosis [17]. IL-10 is an inflammatory suppressor, which can exert an anti-inflammatory effect by inhibiting nuclear factors activity and promoting neutrophil apoptosis to reduce pro-inflammatory factors [18]. Therefore, the levels of CRP, IL-6 and IL-10 were observed in the present study to measure the effects of esketamine on inflammatory response.

Additionally, due to the sedative and analgesic effects of esketamine, the Ramsay sedation score was measured at 5, 10, 20 and 30 minutes after administration of esketamine in this study, which might be one of the reasons for the reduced stress response in the tested group.

**References:**

1. Wan Mohamed Radzi CWJB, Salarzadeh Jenatabadi H, Samsudin N. [Postpartum depression symptoms in survey-based research: a structural equation analysis.](http://pubmed-ncbi-nlm-nih-gov-s.webvpn.njmu.edu.cn:8118/33499833/) BMC Public Health. 2021;21(1):27.

2. Limandri BJ. [Postpartum Depression: When the Stakes Are the Highest.](http://pubmed-ncbi-nlm-nih-gov-s.webvpn.njmu.edu.cn:8118/31670829/)

J Psychosoc Nurs Ment Health Serv. 2019;57(11):9-14. [3. Wszołek K, Żurawska J, Łuczak-Wawrzyniak J, Kopaszewska-Bachorz B, Głowińska A, Pięta B. Postpartum depression - a medical or a social problem?](http://pubmed-ncbi-nlm-nih-gov-s.webvpn.njmu.edu.cn:8118/30513062/) J Matern Fetal Neonatal Med. 2020;33(15):2556-60.

1. Di Florio A, Meltzer-Brody S. [Is Postpartum Depression a Distinct Disorder?](http://pubmed-ncbi-nlm-nih-gov-s.webvpn.njmu.edu.cn:8118/26267038/) Curr Psychiatry Rep. 2015;17(10):76.
2. Stewart DE, Vigod SN. [Postpartum Depression: Pathophysiology, Treatment, and Emerging Therapeutics.](http://pubmed-ncbi-nlm-nih-gov-s.webvpn.njmu.edu.cn:8118/30691372/) Annu Rev Med. 2019;70:183-96.
3. Kroska EB, Stowe ZN. [Postpartum Depression: Identification and Treatment in the Clinic Setting.](http://pubmed-ncbi-nlm-nih-gov-s.webvpn.njmu.edu.cn:8118/32762926/) Obstet Gynecol Clin North Am. 2020;47(3):409-19.
4. Alshammari TK. [The Ketamine Antidepressant Story: New Insights.](http://pubmed-ncbi-nlm-nih-gov-s.webvpn.njmu.edu.cn:8118/33297563/)

Molecules. 2020;25(23):5777.

1. Shinohara R, Aghajanian GK, Abdallah CG. [Neurobiology of the Rapid-Acting Antidepressant Effects of Ketamine: Impact and Opportunities.](http://pubmed-ncbi-nlm-nih-gov-s.webvpn.njmu.edu.cn:8118/33568318/) Biol Psychiatry. 2021;90(2):85-95.
2. Molero P, Ramos-Quiroga JA, Martin-Santos R, Calvo-Sánchez E, Gutiérrez-Rojas L, Meana JJ. [Antidepressant Efficacy and Tolerability of Ketamine and Esketamine: A Critical Review.](http://pubmed-ncbi-nlm-nih-gov-s.webvpn.njmu.edu.cn:8118/29736744/) CNS Drugs. 2018;32(5):411-20.
3. Wang W, Xu H, Ling B, Chen Q, Lv J, Yu W. [Effects of esketamine on analgesia and postpartum depression after cesarean section: A randomized, double-blinded controlled trial.](http://pubmed-ncbi-nlm-nih-gov-s.webvpn.njmu.edu.cn:8118/36451452/) Medicine (Baltimore). 2022;101(47):e32010.
4. Wang W, Ling B, Chen Q, Xu H, Lv J, Yu W. [Effect of pre-administration of esketamine intraoperatively on postpartum depression after cesarean section: A randomized, double-blinded controlled trial.](http://pubmed-ncbi-nlm-nih-gov-s.webvpn.njmu.edu.cn:8118/36862862/) Medicine (Baltimore). 2023; 102(9): e33086.
5. Wong H, Singh J, Go RM, Ahluwalia N, Guerrero-Go MA. [The Effects of Mental Stress on Non-insulin-dependent Diabetes: Determining the Relationship Between Catecholamine and Adrenergic Signals from Stress, Anxiety, and Depression on the Physiological Changes in the Pancreatic Hormone Secretion.](http://pubmed-ncbi-nlm-nih-gov-s.webvpn.njmu.edu.cn:8118/31485387/) Cureus. 2019;11(8):e5474.
6. Kausche FM, Zerbes G, Kampermann L, Müller JC, Wiedemann K, Büchel C, et al. [Noradrenergic stimulation increases fear memory expression.](http://pubmed-ncbi-nlm-nih-gov-s.webvpn.njmu.edu.cn:8118/33358539/) Eur Neuropsychopharmacol. 2021;43:71-81.
7. Colombo A, Giordano F, Giorgetti F, Di Bernardo I, Bosi MF, Varinelli A, et al. [Correlation between pharmacokinetics and pharmacogenetics of Selective Serotonin Reuptake Inhibitors and Selective Serotonin and Noradrenaline Reuptake Inhibitors and maternal and neonatal outcomes: Results from a naturalistic study in patients with affective disorders.](http://pubmed-ncbi-nlm-nih-gov-s.webvpn.njmu.edu.cn:8118/33253437/) Hum Psychopharmacol. 2021;36(3):e2772.
8. Liu H, Lan X, Wang C, Zhang F, Fu L, Li W, et al. [The efficacy and safety of esketamine in the treatment of major depressive disorder with suicidal ideation: study protocol for a randomized controlled trial.](http://pubmed-ncbi-nlm-nih-gov-s.webvpn.njmu.edu.cn:8118/36451150/) MC Psychiatry. 2022 ;22(1):744.
9. Filep JG. [Targeting conformational changes in C-reactive protein to inhibit pro-inflammatory actions.](http://pubmed-ncbi-nlm-nih-gov-s.webvpn.njmu.edu.cn:8118/36465053/) EMBO Mol Med. 2023; 15(1):e17003.
10. Yeh MSL, Poyares D, D'Elia ATD, Coimbra BM, Mello AF, Tufik S, et al. [Sleep characteristics and inflammatory markers in women with post-traumatic stress disorder.](http://pubmed-ncbi-nlm-nih-gov-s.webvpn.njmu.edu.cn:8118/37081449/) BMC Psychiatry. 2023; 23(1):273.
11. Nagata K, Nishiyama C. I[L-10in Mast Cell-Mediated Immune Responses: Anti-Inflammatory and Proinflammatory Roles.](http://pubmed-ncbi-nlm-nih-gov-s.webvpn.njmu.edu.cn:8118/34067047/) Int J Mol Sci. 2021; 22(9):4972.

### **Study goals and objectives:**

To evaluate the effect of single intravenous injection of esketamine on the occurrence of postpartum depression after labor analgesia by observing the incidence of postpartum depression at 1 week and 6 weeks after delivering and side effects such as nausea and vomiting, drowsiness, and nightmares for 48 hours after delivering. To explore the possible mechanisms of the antidepressant effect of esketamine by measuring the levels of NE, E, CRP, IL-6 and IL-10 before labor analgesia , at 24 hours, 1 week, and 6 weeks after delivering.

### **Study design:**

**Participants:** One hundred and twenty primiparous women who underwent labor analgesia from June 1st 2022 to February 28th 2023 in the Affiliated Jiangning Hospital of Nanjing Medical University were enrolled, with single term pregnancy, ASA II, aged 22 to 38. The exclusion criteria were as follows: (1) not suitable for transvaginal delivery; (2) combined with coagulation disorders; (3) having mental disorder; (4) organic or pharmacogenic depression before delivering; (5) combined with functional insufficiency of important organs such as heart, liver, kidney and others. (6) combined pregnancy complications such as hypertension and diabetes; (7) failure of epidural puncture; (8) changed to cesarean section.

**Sample size:** Based on the results of our pre-experiment (10 participants in each group) , the incidence of PPD at 6 weeks after delivering can be reduced by 12% in the tested group. Power analysis showed that a reduction rate of 15% with *α* = 0.05 and a 10% dropout rate within a power value of 90%, a sample size of at least 54 per group were needed. A total of 120 samples were designed in this study, for 60 in each group respectively. There will be a figure which shows the CONSORT flow diagram of the study participant’s recruitment.

**Randomization and allocation concealment:** Participants were randomly assigned to one of two groups. Random tables were generated by SPSS 20.0. One hundred and twenty sealed envelopes were prepared by a statistician who did not participate in the study. The study was performed with neither patients’ nor observers’ awareness of the group to which each patient belonged. To assure concealment of allocation, numbers were kept in sealed and opaque envelopes, which were opened by an anaesthesiologist who was not involved in the study.

### **Methodology:**

**Interventions:** After entering the delivery room, peripheral venous access was opened in all participants, maternal heart rate (HR) , noninvasive blood pressure measurement (NIBP) , electrocardiography (ECG) and pulse oximetry (SpO_2_) were monitored. All women were received oxygen for 3 L/min by transnasal catheter. Epidural anaesthesia were operated between L_2_ and L_3_ after cervical dilation up to 2 ~ 3 cm. After successful puncture, the epidural catheter was placed for 3.5 cm toward the head position and 1% lidocaine was injected for 3 ml. The epidural analgesia pump was connected, in which was 0.75% ropivacaine hydrochloride (20 ml) were diluted by normal saline up to 100 ml. All pumps were set with a bolus of 10 ml, continuous infusion amount of 8 ml/h, single dose of 4 ml, locking time of 15 min and stopped after fetal disengagement. Esketamine (0.2 mg/kg) was intravenously injected after fetal disengagement in Group E, while the equal volume of normal saline was administered in Group C. All anesthesia-related operation were completed by the same anesthesiologist, and the delivering was supervised by the same group of obstetrician. Neither observers nor subjects were aware of the grouping condition, and the pump was configured by an anesthesia nurse who was not aware of the grouping condition as well.

**Main outcome measures:** The incidence of PPD at 1 week and 6 weeks after delivering were evaluated and recorded. The diagnostic criteria for PPD are usually defined in two steps: firstly, Edinburgh Postpartum Depression Scale (EPDS) was used to screen for suspicious patients (score > 9) ; secondly, suspicious patients were strictly tested according to the clinical protocol interview (SCID) ,and the standard for the diagnosis of PPD was consulted by “the Diagnostic and Statistical Manual of Mental Disorders” (DSM-Ⅳ) . The screening and diagnosis were determined by different physicians who did not know the grouping. The Ramsay sedation score before analgesia (T_0_), 5 (T_1_), 10 (T_2_) , 20 (T_3_) , 30 (T_4_) and 60 (T_5_) minutes after administration of esketamine were measured. The scoring criteria were as follows: 1 point, patients were awake, feeling anxiety or irritability; 2 points, patients were awake, being cooperative, directional or quiet; 3 points, patients were awake, only being responsive to commands; 4 points, patients were asleep, being responsive quickly to tapping eyebrow or soft tone stimulation; 5 points, patients were asleep, being responsive slowly to tapping eyebrow or strong acoustic stimulation; 6 points, patients were asleep, being no responsive to tapping eyebrow or strong acoustic stimulation. All women were tested levels of norepinephrine (NE) , epinephrine (E) ,C-reactive protein (CRP) , interleukin-6 (IL-6) and interleukin-10 (IL-10) in peripheral venous blood before labor analgesia (when the woman was quiet in the ward), at 24 hours, 1 week, and 6 weeks after delivering. Serum concentrations of IL-6 and IL-10 were determined by [enzyme](https://baike.baidu.com/item/enzyme/23812841?fromModule=lemma_inlink) linked immunosorbent assay (ELISA) . The volume of postpartum hemorrhage was calculated for 24 hours and side effects such as nausea and vomiting, drowsiness, and nightmares were also recorded for 48 hours after delivering by anesthesia nurses who were not aware of the grouping as well.

**Statistical analysis:** Data analysis was performed by the SPSS 20.0 statistical software package, version 20.0 (SPSS Inc., Chicago, IL, USA). Continuous variables were presented as mean ± SD, and differences between the two groups were analysed with mutual comparison by single factor variance analysis (one-way ANOVA) . The incidence of PPD and side effects were considered as categorical variables, which were presented as n(%) and analyzed with a χ^2^-test (Kruskal–Wallis test). It was considered statistically signifificant since a *p*-value < 0.05.

### **Safety considerations:**

As an anesthetic drug, esketamine was used many years, but there were no sufficient data for the prevention or treatment of psychiatric diseases. Considering the possible risk of hallucinogenic effects and abuse of esketamine, the dose used in this study was slightly lower than the subanesthetic dose, and esketamine was administrated only once. If side effects such as nausea, vomiting, drowsiness, and nightmares in 48 hours after the operation, free treatment will be provided by department of Anesthesiology, the Affiliated Jiangning Hospital of Nanjing Medical University.

### **Follow-up:**

In this study, all participants were followed up for six weeks after labor analgesia, although side effects were recorded for 48 hours after delivering.

### **Data management, statistical analysis and quality assurance:**

The Consolidated Standards of Reporting Trials (CONSORT) recommendations are followed in this study. The recruitment of volunteers, calculation of sample size and unblinding and so on have been explained above. We have special quality controllers, data recorders, evaluators, statistical analysis indicators etc. In addition, we have subject reception room and archival storage room in which all date of subjects are preserved.

### **Expected outcomes of the study:**

We predict that esketamine used in women underwent labor analgesia can significantly reduce the incidence of postpartum depression at 1 week and 6 weeks after delivering without increasing related side effects. Effect of its antidepressant may be related to the reduction of stress response and inflammation.

### **Duration of the project:**

01/06/2022-31/07/2022: Scheme discussion and proposal report;

01/08/2022-28/02/2023: Clinical trials and data collection;

01/03/2023-31/03/2023: Data analysis and discussion of the results;

01/04/2023-01/05/2023: Paper writing and submission.

### **Project management:**

This work was supported by Department of Anesthesiology, the Afﬁliated Jiangning Hospital of Nanjing Medical University,

1. The project leader is the corresponding author, who is fully responsible for the project and has the right of management.
2. Once the work of the members of the research group is determined, it shall not be changed without approval.
3. All data formed in the research work shall not be in short supply and be existing. It shall not be copied or published without permission.
4. The experimental records should be timely, accurate, true and complete.
5. The research group should report the phased summary, evaluation and work process to the Science and Education Department on time.
6. After all the research work is completed, the project leader will write the summary report.
7. The research cost, results and archives management all follow the scientific research regulations of the institute.

### **Ethics and informed consent:**

The study was performed in accordance with the [declaration of Helsinki](https://www.wma.net/policies-post/wma-declaration-of-helsinki-ethical-principles-for-medical-research-involving-human-subjects/). Ethical approval for this study (2021-03-031-K01) was provided by the Institutional Ethics Committee of the Affiliated Jiangning Hospital of Nanjing Medical University. All volunteers involved were informed of the proposal and gave their written, informed consent.

**Funding:**

No funding was received for conducting this study and this work was supported by departmental funding only.

### **Curriculum Vitae of investigators:**

corresponding author: Wei Wang, male, was born in June 1985. MD, associate chief physician, lecturer of Nanjing Medical University, member of the first and second Youth Committee of Anesthesiology Branch of Nanjing Medical Association, amateur editor of Clinical Anesthesiology.

Working in the department of Anesthesiology in the Affiliated Jiangning Hospital of Nanjing Medical University from August 2007 to now.

Receiving the master degree of anesthesiology from Nanjing Medical University in December, 2013.

Receiving the MD degree of joint training clinical medicine from Nanjing Medical University in December, 2019.

As the principal, having participated in a number of clinical and basic researchs.

As the first author or corresponding author, having published several papers, 6 of which were included in SCI.
